# Chemical–physical and dynamical–mechanical characterization on *Spartium junceum* L. cellulosic fiber treated with softener agents: a preliminary investigation

**DOI:** 10.1038/s41598-020-79568-5

**Published:** 2021-01-08

**Authors:** Giuseppina Anna Corrente, Francesca Scarpelli, Paolino Caputo, Cesare Oliviero Rossi, Alessandra Crispini, Giuseppe Chidichimo, Amerigo Beneduci

**Affiliations:** Department of Chemistry and Chemical Technologies, University of Calabria, Via P. Bucci, Cubo 15D, 87036 Arcavacata Di Rende, CS Italy

**Keywords:** Physical chemistry, Materials chemistry

## Abstract

Long cellulose fiber (10–30 cm), extracted from *Spartium junceum*, was chemically treated with different softening agents with the aim to improve its textile applicability. A preliminary sensory evaluation of the treated fibers revealed an evident, though qualitative, improvement of the fiber softness. The effects of the softening agents on the fiber was evaluated quantitatively, by means of macroscopic measurements of the wettability, viscoelasticity, and thermal (thermal gravimetry) properties. Moreover, the effects of the softening treatments on the microscopic structure of the fiber and on its properties at a molecular level, were studied by optical and scanning electron microscope and X-ray diffraction (XRD), respectively. The macroscopic analysis showed that the softeners used increases the hydrophilicity and water wettability of the cellulose fiber with respect to the raw one. Moreover, the dynamical mechanical analysis on sample yarns showed that the softeners increase the interfiber frictional forces. A linear correlation between the interfiber friction and the increase of hydrophilicity and fiber wettability was shown. The treated fiber exhibits a more homogeneous thermal behaviour, due to more homogeneous structural features, since the thermal-induced cellulose fibrils depolimerization undergoes a marked temperature range contraction. These data can be well related with those obtained by microscopy analysis, showing that the fiber surface, after the treatment, appears thinner and less rough, as well as with the XRD analysis, which shows that softeners induce a significant decrease of the fiber crystallinity.

## Introduction

In the last decades there was an increasing world trend towards the maximum utilization of recyclable resources and the attention to an eco-sustainable and environmentally friendly development. In the textile sector, there is still a strong interest on the development and production of chemical synthetic fibres based on non-renewable fossil-fuels, especially for advanced technological applications^1,2^. However, the increasing production costs of petroleum-based materials, and the costs associated to their environmental impact, have boosted the need for using renewable natural fibres. Indeed, natural fibres derived from plants, rich in lignin, hemicellulose and cellulose, can be used as precursors or components in several advanced materials, such as renewable plastics, polymer blends, additives and solvents^3–5^. These bioproducts can be used for various industrial sectors such as bio-buildings, furniture, automotive, packaging, etc. as well as for the textile sector.

As far as the textile sector is concerned, cotton and linen are the most important natural fibers. However, their production requires large land usage at the expenses of other food crops. On the other hand, as the food demand is increasing, we have to search for sustainable food productions that do not heavily impact on the ecosystems^6^. To this aim, one of the green approaches to the natural fiber textile industry, is to extract fibers from crops by-products such as cornhusks^7^, transforming thereby wastes into resources. Unfortunately, this approach is not so straightforward since the fibers obtained are, generally, not comparable to cotton and linen, in terms of production costs and performances.

Another approach is to extract fibers from rapidly and spontaneously growing shrubs such as Spanish broom (*Spartium junceum* L., SJ), which is also endemically distributed almost in all over the world. The lignocellulose extracted from SJ has been used for the adsorption of mercury from water^8^. Moreover, the cellulose extracted from SJ has been surface functionalized with hydrophobic groups in order to be suitable as adsorbent material for the successful removal of petroleum hydrocarbons and bisphenol A from water^9–11^. Fabrics made of SJ cellulose were also surface functionalized with TiO_2_ and used in photocatalytic bacterial inactivation^12^. Additionally, functionalized SJ cellulose has been used as natural reinforcement in polymer composites^13,14^. Highly flexible and mechanical resistant cellulose fiber with length up to 40 cm, can be extracted from the so-called vermenes of the plant, the branchlets, by mechanical processes, after a preliminary maceration process with bacteria (retting)^15^ or alkali^14,16–18^. In principle, due to the large availability of Spanish broom, especially in the Mediterranean area and in hot and dry climate regions, and the attractive properties of its fiber, it could become a main natural source of cellulose fiber for textiles and of lignocellulose for other applications, provided if the fiber extraction process would be efficient, inexpensive and scalable. To this aim, we are developing an automated production process based on an alkaline pre-treatment of the shrub in a 5% (w/w) of sodium hydroxide water solution at 80 °C, followed by the separation of the cellulose fiber by means of specific brushes that mechanically harvest the long fibers from the shrub. A first pilot plant, already operative at the University of Calabria, can produce about 0.6 kg/h of fiber having a 95% (w/w) of cellulose. It must be considered that the fraction of cellulose fiber that can be extracted from the vermenes is of the order of 10% (w/w) on a dry basis over the entire vegetable mass processed. Industrial scale up is underway and it is expected that the final extraction plant should allow the production of up to 6–10 kg/h.

The characteristics of the extracted fiber are very important to obtain a high quality product. In the textile field, the fibers must have certain properties in order to be transformed into thin and flexible yarns and then into fabrics. Fiber quality is influenced by the length, which can vary from fiber to fiber, determining its quality, fineness and thickness. The longer and thinner is the fiber, the higher is its quality. The fiber linear mass density, which measures the weight of the fiber in relation to its length, is an important metrics for fiber finesses and can be expressed in Denier (den, g/9 km) or tex (g/km). Additional properties are brightness, hygroscopicity, breathability and mechanical properties. The above parameters can be improved by softening methods. Softening can be performed by mechanical, biological or chemical treatments. The aim of the present study was to investigate the effect of different softening agents on the physico-chemical, morphological and mechanical properties of the fiber.

## Results and discussion

**Figure 1:**
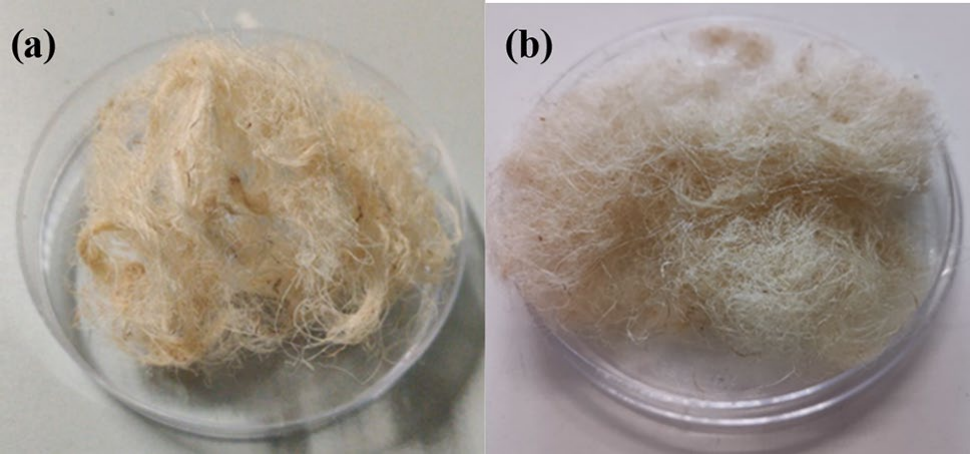
Representative samples of the raw (**a**) and treated cellulose fiber with the chemical softener TTAB (**b**).

The study of the morphological, structural, thermal and mechanical properties, before and after chemical treatments, is of noteworthy importance for the evaluation of the effects induced by the chemical treatment with the softeners. From preliminary tactile and visual observations (Fig. 1) carried out by comparison of the treated fiber with the raw fiber, it was possible to observe that the treated one appears to be softer than the raw fiber.

This qualitative evaluation was followed by a quantitative assessment at a macroscopic, microscopic and molecular level of the effect of the softeners on the fiber.

### Morphological characterization

**Figure 2:**
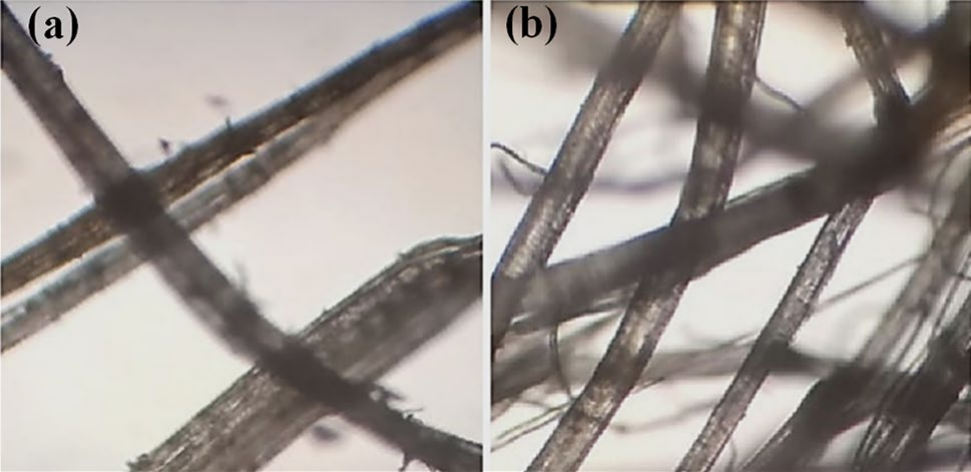
Representative optical microscopy images (100×) of the raw fiber (**a**) and treated cellulose fiber with the chemical softener TTAB (**b**).

The effects of the chemical treatments on the fiber morphology has been investigated by optical and SEM. Figure 2 shows representative optical images of the raw and treated fibers. It can be observed that the lateral spikes characteristic of the pristine fiber, which confer a certain degree of roughness of its surface, are no longer present after the treatment.

**Figure 3:**
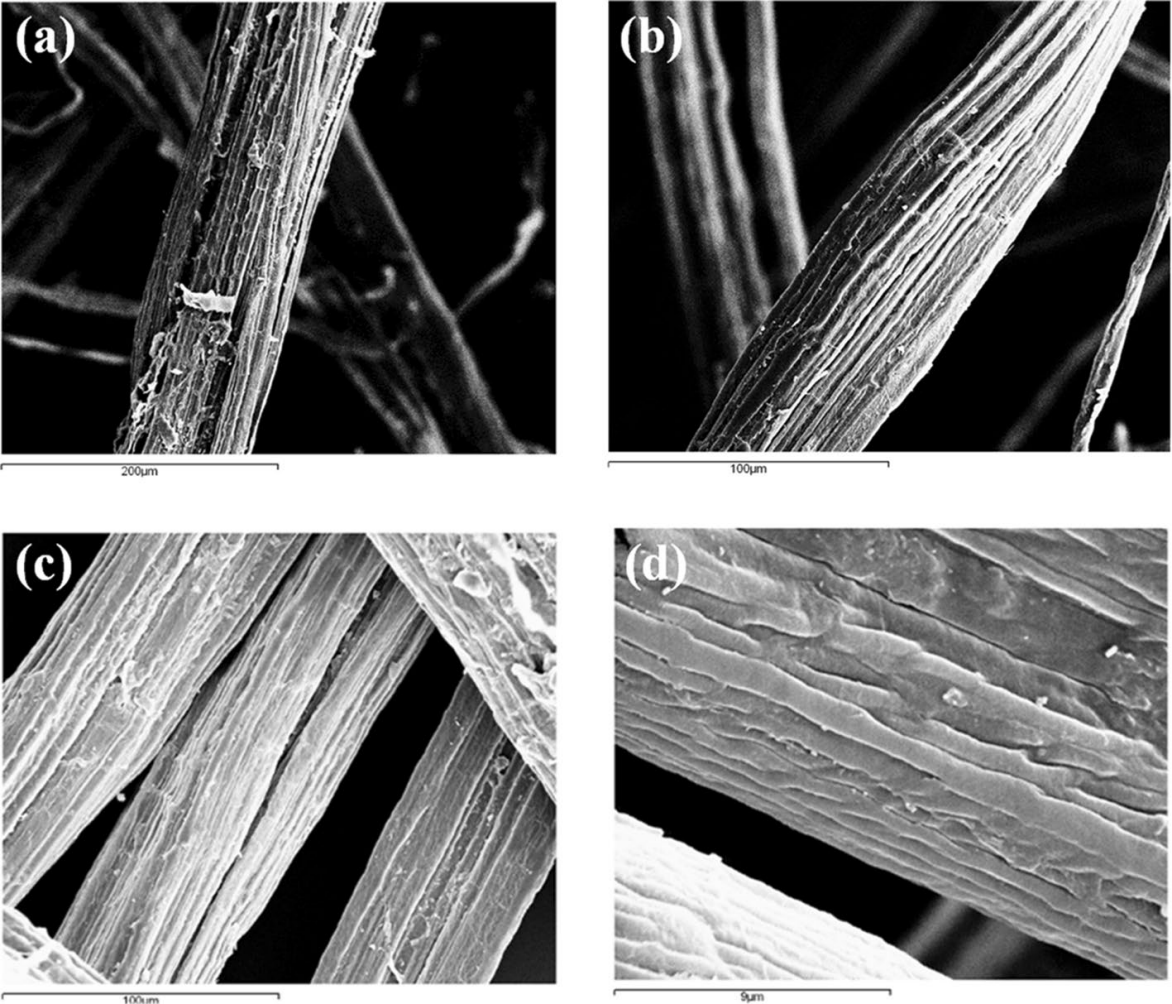
SEM microphotographs of the raw fiber (**a**) and of the fibers treated with the chemical softeners TTAB (**b**), LP (**c**), and LS (**d**).

The ultrastructural surface morphology of the cellulosic fiber was examined using a SEM. The surface observation of the raw fiber reveals the typical structure of cellulose, organized in aggregated microfibrils with sub-micrometer size diameter, to form fibrous structures of cylindrical cross-section, called bundles (Fig. 3a). The comparison between the SEM images acquired on the treated fiber (Fig. 3b–d) and the raw one, does not allow to highlight specific effects of the different softeners on the structural morphology of the fibers. Therefore, the chemical softeners do not de-structure the typical fiber organization.

However, the softener treatment induces a significant reduction of the bundles diameter, as reported in Table 1, that collects the results of the average diameter of the raw and treated fibers. The fiber diameter populations related to the raw and the treated fiber samples are all distributed normally at the 0.01 confidence level. Therefore, in order to ascertain whether the four populations have significant different mean diameter, a One-Way ANOVA statistical test was used. It shows that the fibers treated with the softeners have a mean diameter significantly different from that of the raw fiber (at the 0.01 confidence level). In addition, the statistical test shows that there is not any difference among the mean diameters of the three treated sample populations (at the 0.01 confidence level).

**Table 1:** Diameters of the raw and the treated cellulose fiber bundles.

Sample	Diameter (μm)			
	Max	Min	Mean^a^	SD
Raw fiber	200	50	124	44
TTAB	70	35	55^b^	11
LP	105	40	69^b^	18
LS	78	40	57^b^	11

^a^Averaged over 60 fiber bundles diameter determination on the full data set coming from the same softener at room temperature (irrespective of concentration and time).

^b^Mean value significantly different with respect to that of the raw fiber at the 0.01 level (one-way ANOVA test).

An important parameter in textile application is the fiber fineness. From the thickness of the single fiber filament it is possible to determine the fineness of the fiber, *i.e.*, its linear mass density. For the raw fiber, the linear mass density was determined to be 4.7 (± 0.4) tex. The effect of all the softener treatments was to decrease the fiber linear mass density to an average value of 2.1 (± 0.6), thus significantly increasing the fiber fineness.

### PXRD analysis

Cellulose, the main fiber component, is a semicrystalline polymer, characterized by crystalline and amorphous regions separated by not well defined boundary lines^19^. The crystalline areas, which present parallel polymer chains held together by hydrogen bonds and Van der Waals interactions, are responsible for the high tensile strength of cellulose fibers^20^. Since the fiber rigidity, and therefore its processability, is related to the cellulose crystallinity, the reduction of the crystalline portion, with consequent increase of the amorphous region, implies an increase of the fiber softness. A reduction of the fiber crystallinity can be obtained through the use of a softener, since, according to Igarashi et al., the softening effect of a fabric softener could be ascribed to the loss of some hydrogen bonds and Van der Waals interactions between the ordered polymeric chains, probably due to the “insertion” of softeners micelles^21,22^. On this basis, the measurement of the Crystallinity Index (CI) could represent an important parameter for assessing the fiber workability (fiber strength and elasticity). Moreover, the amorphous regions provide accessible volume for water thus contributing to the increase of the hygroscopicity of the fiber^15^.

**Figure 4:**
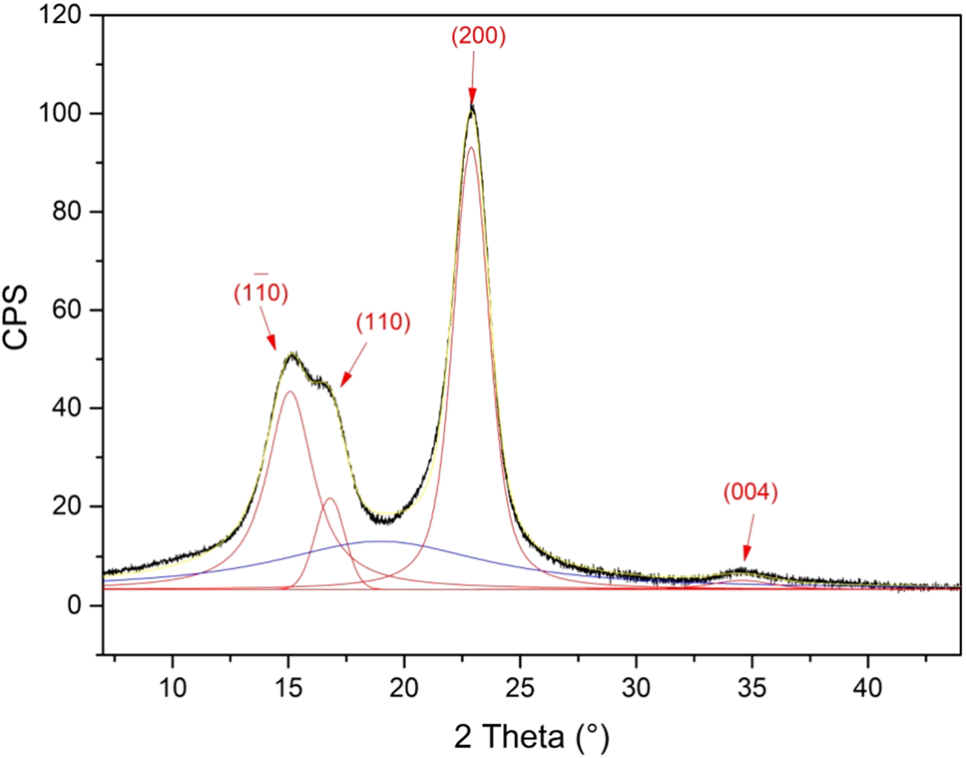
XRD profile of the raw fiber with its deconvolution curves (crystalline peaks: red lines; amorphous region: blue curve; cumulative fit curve: yellow line).

The CI of the raw fiber and that of the softened samples was determined by the PXRD profiles deconvolution using the Voigt function (results reported in Table 2). The PXRD pattern of the raw fiber, together with the individual peaks extracted by the Voigt fitting procedure, is reported in Fig. 4. The deconvolution process allowed to identify four crystalline peaks of cellulose (1ī0, 110, 200, 004 at 15.0°, 16.8°, 22.9° and 34.4°, respectively; red curves)^23^ and to calculate CI from the ratio between the area of the four crystalline peaks and the total area of the pattern. The calculated CI value of 62% is in line with those already observed for Spanish Broom cellulose fibers^18,24,25^ and for other commercial fibers^7^.

The effect of the different softeners used and the influence of experimental parameters, as time, temperature and additive concentration, was studied in terms of CI variation.

When the softener TTAB was used at a concentration of 100 mg/L and the modified fiber was treated for 60 min at room temperature, a reduction in crystallinity of 10% was recorded (Table 2). The effect of the temperature variation was subsequently evaluated treating the fiber at 40 °C with the same softener concentration and for the same time; in this case, a CI value (51.6%) similar to that observed at room temperature (50.1%) was recorded, demonstrating that the temperature has little influence on the TTAB-based softening process. Hence, with a view of the industrial scaling-up of the process, the treatment at r.t. may be preferred. The effect of the softener concentration was then investigated treating the fiber for 60 min at r.t. with different TTAB concentrations (200 mg/L, 50 mg/L and 10 mg/L). Interestingly, the increase of TTAB concentration from 100 mg/L to 200 mg/L seems to affect negatively the process, since the use of a higher TTAB concentration leads to a significantly lower CI decrease (4.5%) with respect to the 100 mg/L concentration. On the contrary, the use of a very low TTAB concentration (10 mg/L) determines a higher crystallinity reduction (14.4%) with respect to the 100 mg/L concentration. The effect of the contact time (60, 30 and 15 min) was finally assessed both at room temperature and at 40 °C; in both cases, the best result was obtained at the shortest time (15 min). Thus, the greatest decrease of the sample crystallinity (21.4%) with respect to the raw fiber can be reached using a concentration of TTAB softener of 10 mg/L, at room temperature and for 15 min as treatment time.

When the softener LP was used at r.t. for 60 min at concentration of 100 mg/L, a negligible reduction in crystallinity (0.5%) was observed. The treatment with a higher softener concentration (200 mg/L), at the same temperature (r.t.) and for the same time (60 min), was subsequently carried out. In this case, a higher CI decrease (11.3%), with respect to the lower concentration process, was recorded, proving that, differently than TTAB, the softening effect of LP requires a high softener concentration. An increase of temperature (60 °C) at the same concentration condition and contact time, does not affect the degree of crystallinity with respect to room temperature. Therefore, similarly to the TTAB-process, the increase in temperature does not benefit the softening process. The effect of the contact time was then established at the best softener concentration, that is 200 mg/L. In this case, the best softening effect is reached at the intermediate treatment time of 30 min, where a CI value of 48.3% is calculated.

Using LS as softener, while a modest decrease of crystallinity (8%) was recorded at 100 mg/L of concentration and treating the fiber for 60 min at r.t., an increase of concentration benefits the softening process, resulting in a significant CI reduction (19%) (Table 2). However, when the temperature is increased up to 60 °C, keeping the same concentration of 200 mg/L, a negligible CI decrease (2.1%) was found, demonstrating that temperature variation negatively affects the LS softening process. Finally, fixing the best condition of concentration and temperature (200 mg/L and r.t.), the effect of the treatment time was checked. Even on the case of the LS softener, the higher crystallinity loss (20.9%), with respect to the raw fiber, was obtained with intermediate treatment time (30 min).

**Figure 5:**
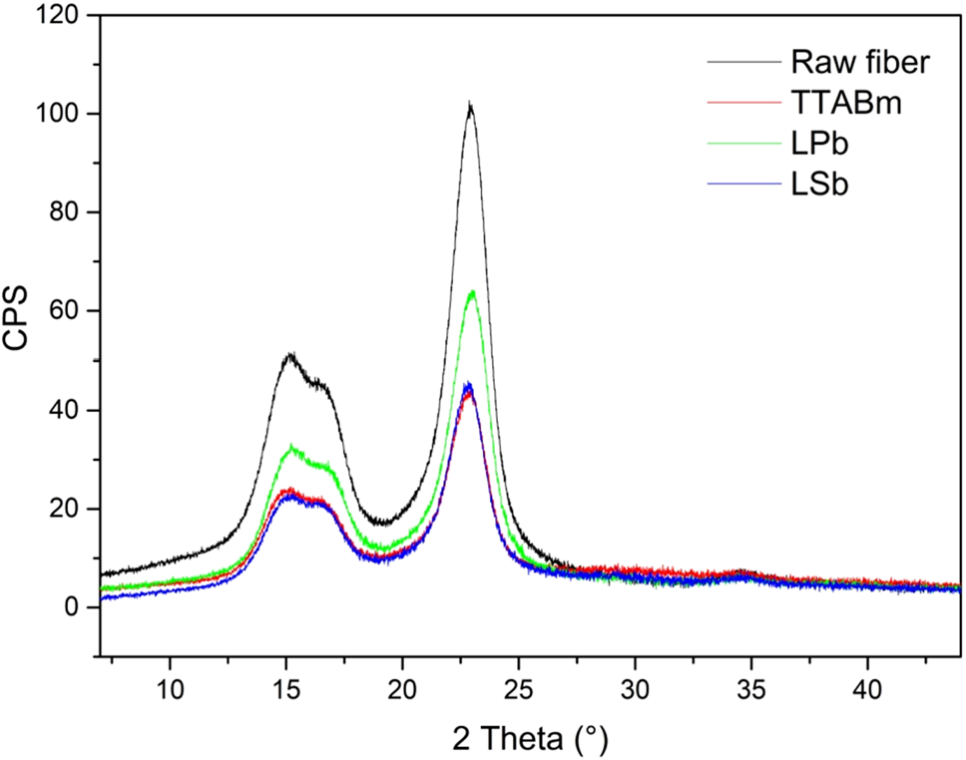
PXRD patterns of the raw fiber, sample TTABm, sample LPb and sample LSb.

The PXRD pattern of the fiber treated with the softener TTAB, LP and LS, at the best conditions for each of them (TTABm, LPb and LSb) and compared to the diffraction profile of the untreated fiber, are reported in Fig. 5. The greatest loss of crystallinity is obtained when the fabric softener TTAB is used at a very low concentration, with the fastest process and at room temperature, representing a great advantage from an industrial point of view.

**Table 2:** Crystallinity Index of the Spanish broom fiber treated with different softeners and under different experimental conditions.

	Experimental conditions			Crystallinity Index %
	Concentration (mg/L)	Temperature (°C)	Time (min)	
**TTAB**				
a	200	r.t	60	57.5
d	100	r.t	60	50.1
e	100	40	15	46.5
f			30	50.9
g			60	51.6
l	50	r.t	60	52.0
m	10	r.t	15	40.6
n			30	45.9
o			60	47.6
**LP**				
a	200	r.t	15	55.0
b			30	48.3
c			60	50.7
d	200	60	60	52.0
e	100	r.t	60	61.5
**LS**				
a	200	r.t	15	44.0
b			30	41.1
c			60	43.0
d	200	60	60	59.9
e	100	r.t	60	54.0

It is worth to note that, for all three softeners, the increase in temperature is not advantageous to the softening process, since the best results were obtained at r.t. Moreover, fast treatment times are preferable to the 60 min treatment. The greater softening action in shorter times could be ascribed to a surfactant-removal effect probably occurring for longer treatment times. The effect of concentration, on the other hand, depends on the softener used; indeed, the best working concentration for TTAB corresponds to 10 mg/L, whereas the softening effect of LP and LS requires higher concentrations (200 mg/L).

In the attempt to understand the different effect of the concentration with respect to the type of softener, the mechanism of micelles formation can be invoked. However, although the tested LS concentrations are above its critical micelle concentration (CMC) in water^26^, the best working concentrations for TTAB and LP (10 mg/L and 200 mg/L, respectively) are far from their CMC^27–29^. Then, surely for both TTAB and LP the mechanism of micelles can be excluded.

Additionally, the concentration of 10 mg/L for TTAB is far from the concentration conditions necessary for the induction of micellization or aggregation on the cellulose surface, so far observed for nanocrystalline cellulose^27,30,31^ and cellulose^32^. Therefore, at these concentrations, electrostatic interactions can come into play between TTAB and cellulose, rather than the hydrophobic ones, supported by the ionic nature of the TTAB surfactant^27,28,30,32^. However, it is difficult to explain the observed decrease of CI with decreasing TTAB concentration with the available data. It seems that at low concentration disordering effects predominate, while, when concentration increases other processes come into play, such as the formation of ordered networks between cellulose and the ions of the surfactant. This interesting phenomenon deserves further investigation, by extending the concentration range up to the micellar one and addressing the correlation between the degree of cellulose crystallinity and the interaction mechanism between the cellulose surface and TTAB.

### Thermal analysis

**Figure 6:**
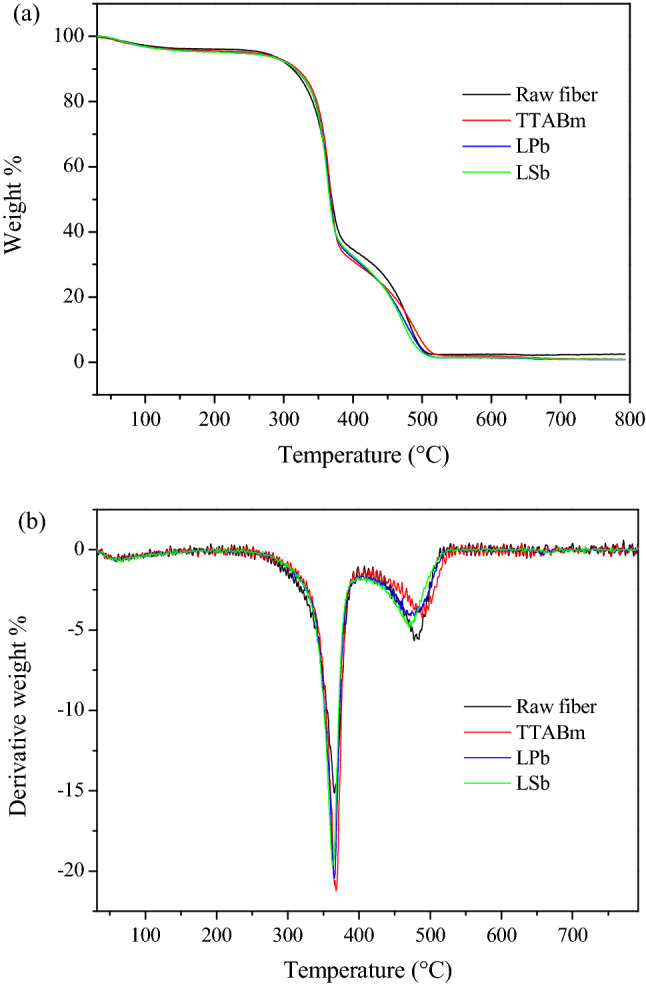
TG curves (**a**) and DTG curves (**b**) of the raw fiber and TTABm, LPb and LSb samples.

**Figure 7:**
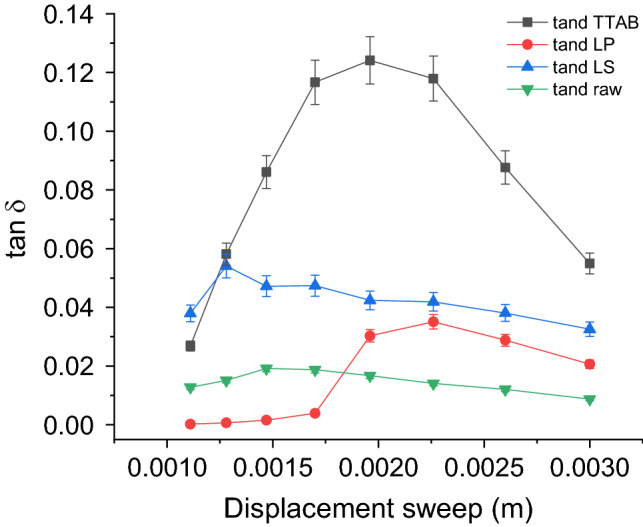
Elastic E’ and viscous E’’ moduli as a function of an increase in the deformation applied (displacement sweep).

The effect of the three different softeners on the extracted fiber was investigated further by analysing the thermal behaviour. Actually, although fiber chemical modifications, easily detectable by TGA, do not seem to be induced by the use of softeners, changes on the packing of the polymeric chains, reflecting on the polymer crystallinity, can also determine a different thermal behaviour of cellulose^33^. In particular, thermogravimetric analysis (TGA) was carried out on the raw fiber and on samples TTABm, LPb and LSb, which present the greatest loss of crystallinity, thus chosen as representative samples for each softener used. The TGA and DTG curves are shown in Fig. 6a,b, respectively, and the TGA data are reported in Table 3 and Table S2. The thermogravimetric analysis shows that the raw fiber decomposition reactions occur in three main steps at maximum temperatures (T_max_) of 60, 365.4 and 483.9 °C (Fig. 6a,b, black lines; Table 3 and S2). The first transition corresponds to the moisture loss; the second step, which is accompanied by the greatest weight loss (53.7%), is related to the cellulose depolymerization, which is influenced by the polymeric chains packing, followed by chain scission, dehydration and decarboxylation reactions; lastly, the third transition, which is partially superposed to the second, consists in the charred residue decomposition and in the lignin pyrolysis^34–36^. The treated fibers thermograms are similar to the raw fiber TGA curve (Fig. 7), showing the same three transitions at similar temperature values; the TG curves similarity confirms that the softeners used do not chemically modify the polymer. Indeed, as already observed by Hideno^37^, the thermal behaviour of amorphous cellulose is comparable to that of crystalline cellulose. However, slight variations in the onset temperature (T_onset_) and in the transition temperature range relative to the second and third transitions are present (Table 3 and S2). Since the main decomposition process of cellulose, the one influenced by its crystallinity, takes place in the second step, this will be the only one taken into consideration. In particular, the second thermal step of the softened samples, despite showing a slightly higher T_ons_, occurs in a temperature range of about 10 °C narrower than the untreated fiber. Then, the greater structural homogeneity of the treated samples, presenting reduced separation between amorphous and crystalline areas, could be responsible of the faster second thermal process. Moreover, in the thermal curves of the treated fibers, the DTG peak relative to this transition appears more intense, thus associated to weight loss between 0.7–4.1% greater than the raw fiber; in addition, this peak appears sharper than that observed for the more crystalline pristine fiber, as already reported by Hideno^37^. These findings indicate an increased thermal susceptibility of the softened samples, probably due to a reduction of hydrogen bonds and Van der Waals interactions, in agreement with the PXRD analysis results.

**Table 3:** Thermogravimetric data relative to the second thermal transition for the raw and TTABm, LPb and LSb samples.

Sample	Transition temperature range (°C)	Inflection point (peak max) (°C)	Onset (°C)	Weight loss (%)
raw fiber	256–407	365.4	340.7	53.7
TTABm	268–409	365.3	350	57.8
LPb	261–400	365.3	346.7	55.2
LSb	264–398	363	345.2	54.4

### Wettability

The study of materials wettability is of noteworthy importance in this field of research since it can give information on the effect of a chemical treatment on the hydrophilic/hydrophobic nature of a material^38,39^. Therefore, hydrophilicity and hydrophobicity are closely related to wettability, that is the intimate molecular contact between a liquid and a solid surface. This surface parameter was evaluated by measuring the maximum pull at the water/fiber/air interface, similarly to the de Noüy ring and Wilhelmy plate methods, but using the fiber as a probe. The pull tension is correlated to the surface (or adhesion) tension (σ) of the liquid put in contact with the probe, and can be generalized by the Eq. (1)^40–42^.

1 $$ T_{\max } = f \sigma $$

where $T_{max}$ is the maximum pull tension measured by the microbalance, $\sigma$ is the surface tension of pure water and *f* is a geometric parameter accounting for the geometry of the probe and the shape of the interface, which is determined by the surface material properties of the probe used. Therefore, $T_{max}$ is essentially a function of the fiber surface energy and the water contact angle on the different fiber probes, being the other conditions the same, i.e. the geometry of the probe and the liquid. Thus, higher pull tension means higher adhesion of the liquid water to the probe surface and therefore, higher wettability (higher hydrophilicity of the fiber).

Table 4 collects the relative pull tension values measured with probes made of fiber treated with the selected different softeners referenced to that measured on the raw fiber. Since the measured values are found to be independent of time and temperature of treatment, only the values obtained from different softeners are shown in terms of average.

**Table 4:** Wettability of the fibers treated with different softeners expressed as relative pull tension and water contact angle (WCA).

Sample	Average pull tension (mN/m)^a^	SD	Average WCA^a^ (°)	SD
Raw	0		99	2
TTAB	5.1	1.7	73^b^	14
LP	1.9	0.3	86^c^	9
LS	2.4	0.4	75^d^	5

^a^Average values over the full data set coming from the same softener used (irrespective of concentration, temperature and time).

^b^Significantly different with respect to the raw fiber at the 0.01 level (one-way ANOVA test).

^c^Significantly different with respect to the raw fiber at the 0.5 level (one-way ANOVA test).

^d^Significantly different with respect to the raw fiber at the 0.05 level (ONE-WAY ANOVA TEST).

For all the treated fibers there is a significant increase of the average pull tension with respect to that measured for the raw one, thereby indicating a higher affinity of water for the treated fibers. Consequently, the softened fibers are more hydrophilic than the raw one.

The fiber wettability was also evaluated by measuring the water contact angle (WCA). Table 4 reports the average contact angle for the raw fiber and for the fibers treated under different softening conditions. The value obtained for the raw fiber is slightly larger than that of Spanish broom cellulose obtained previously by measuring it on stretched fibers^9^ rather than on pellets. Spanish broom cellulose shows, in general, a WCA larger than that of pure cellulose (40°)^43^, likely because its extraction by the alkaline digestion from the vegetable does not completely remove the lignin (from 2 to 5% of lignin is always found after extraction and subsequent washing). However, the data collected in Table 4 clearly show that the WCA of all the fibers treated with the different softening agents is smaller than that measured for the raw fiber, indicating that they have a more hydrophilic surface, accordingly with the previous experiment on the pull adhesion tension.

### Viscoelastic properties

DMA analysis of fiber was proposed as a method to obtain information on the interfiber friction, arising from the interaction forces of attraction/adhesion between the fiber surfaces in contact and/or by the mechanical ploughing action due to surface roughness^44^. When a fibrous structure is subjected to cyclic loading, the energy loss due to friction can be evaluated by measuring the storage (E’) and the loss modulus (E’’) which are related by the loss factor, $tan\delta = E^{\prime \prime } {/}E^{\prime }$, where $\delta$ is the phase angle between stress and strain resulting from the viscoelastic nature of the material. When a yarn is subjected to cyclic loading, the frictional energy loss is due to the contribution of the intermolecular friction due to the polymer chains constituting the individual fibers and to the interfiber friction, which is generally the dominant force^44^. Interfiber friction depends on the cyclic loading conditions (frequency, load, gauge length, staple length), on the twist level of the yarn (i.e., the number of twist/cm), and on the nature of the fibers^45^. It is especially affected by finishing agents which change the fiber surface properties^46^. Therefore, under the same experimental conditions and the same twist level, any difference in the frictional energy loss among fibrous samples, is mainly due to the different surface properties of the fibers. Here we used DMA analysis to verify whether the softening treatment affects the frictional forces between Spanish broom cellulose fibers leading to different viscoelastic behaviour.

Figure 7 shows the $tan\delta$ as a function of the displacement for the raw yarn and the yarns treated with the different softeners. Since we did not observe any significant difference among the samples treated with the same softener under different conditions (Table S1), for each softener, each point in the graph is the average value calculated over all the treatment conditions using that softeners.

Figure 7 clearly shows that the softening treatments increases the interfiber friction with respect to the control yarn made by the raw fibers, which has the lowest $tan \delta$ across the whole displacement. The sample yarn treated with the TTAB softener has the highest $tan\delta$ value while, the other two samples show intermediate frictional energy loss. This finding mostly matches well with the following decreasing hydrophilicity trend (pull tension and WCA) of the fibers, TTAB > LS ≥ LP > Raw (Table 4).

**Figure 8:**
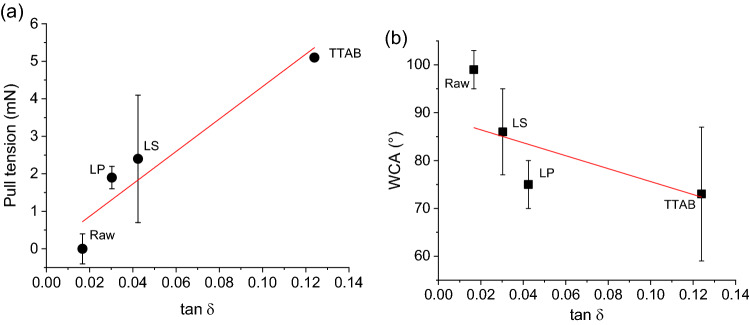
Correlation analysis between fiber-on-fiber frictional energy loss and fiber surface hydrophilicity.

Indeed, the energy loss due to friction ($tan\delta$) is positively correlated to the pull tension (Fig. 8a; Pearson’s r = 0.97; R^2^ = 0.95) and fairly well negatively correlated to the WCA (Fig. 8b; Pearson’s r =  − 0.80; R^2^ = 0.64).

## Conclusion

The effects of different commercial softeners on the cellulose fiber extracted from *Spartium junceum* L. were evaluated. Chemical softening, which affects the stiffness and roughness of the fiber, is an important pre-treatment process used to improve the fiber quality, which can be then more easily transformed into high linear density yarns. In this work, we reported the use of three different softeners belonging to different chemical classes and these were managed in different conditions (concentration, temperature and time of treatment). The preliminary sensory evaluation of the fibers, performed in order to express soft feeling of the treated fibers versus the raw one, revealed that the softeners employed do effectively increase the fiber softness. The PXRD structural analysis showed that the softeners induce a significant reduction of the fiber crystallinity that mainly depends on the softener concentration and the contact time, while it is quite independent on the temperature. Interestingly, for TTAB the highest crystallinity reduction was observed at low concentrations and at short contact times, in contrast to the other softeners, that require higher concentrations and longer times. The significant reduction of the crystallinity index (as high as 20%) induced by the softening agents, clearly indicates that the softeners interact with the cellulose fiber by affecting the interchain hydrogen bonding network, which is responsible of the ordered structure of cellulose crystalline domains. These structural changes can be explained by the adsorption of the softener molecules not only onto the fiber surface but, also, in the fibril inside. Indeed, it was reported that, in the case of ammonium quaternary salts, the softener is distributed in the whole cross section of the fiber (cotton) being mainly accumulated in the lumen (fiber interior) and in the crenulations, rather than on its surface and secondary wall^47^.

We believe that his adsorption is also the cause of the significant mean fiber diameter reduction, the increase of the hydrophilicity and the increase of the loss tangent. We showed that these effects depend on the nature of the softener, even though a correlation with the working conditions could not be made, as done for the XRD analysis. Therefore, if on one hand, the softeners induce a marked crystallinity reduction of the cellulose fiber, pointing toward a softening effect, they also cause a significant increase of the interfiber frictional force that is correlated to the increase of the fiber hydrophilicity. Even though, the softening mechanism is still not yet clarified, the last two results are in contrast to the main hypotheses so far suggested. One is that the softener molecules reduce the friction between adjacent fibers because they form a structured hydrophobic layer on the cellulose surface by exposing their alkyl tails toward the air after the drying process^21^. The other one is that the softeners prevent the formation of cross-linking bound water layers between the fibers during the drying process, which otherwise increases the fiber stiffness^21,22^.

However, it must be highlighted that the softener concentrations used in this study are far below those conventionally employed, and this may explain the discrepancy. At the same time, it highlights the complexity of the softener/cellulose interaction that might involve several different steps depending on the concentration regime.

## Methods

### SJ fibres extraction process

Cellulose fiber was mechanically separated from the vermenes previously macerated under alkaline conditions (5% w/w NaOH) at 80 °C for 30 min. Before the softening treatment, the extracted cellulose was washed with water several times, combed and left to dry at room temperature.

### Softening agents

**Figure 9:**
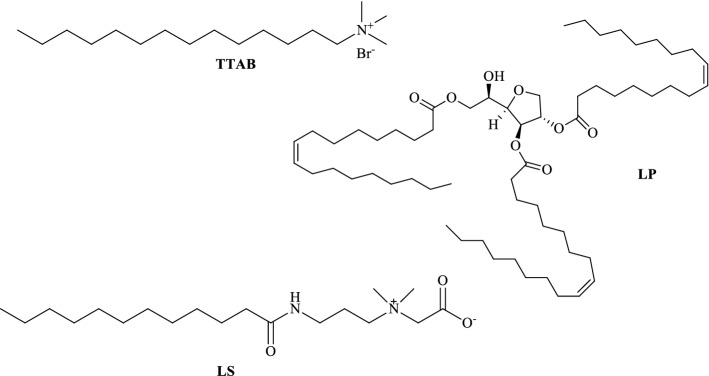
Molecular structures of the softeners used: Tetradecyltrimethylammonium bromide, TTAB, (Z)-(2R,3R,4S)-2-((R)-1-hydroxy-2-(oleoyloxy)ethyl)tetrahydrofuran-3,4-diyl dioleate, LP, 2-((3-dodecanamidopropyl)dimethylammonio)acetate, LS.

The softening agents used were the tetradecyltrimethylammonium bromide (TTAB) (Sigma Aldrich), the commercial softener Lauropan S/85 (LP) whose conditioning agent is the (Z)-(2R,3R,4S)-2-((R)-1-hydroxy-2-(oleoyloxy)ethyl)tetrahydrofuran-3,4-diyldioleate and the commercial softener Laucosol B45 (LS), whose conditioning agent is the 2-((3-dodecanamidopropyl)dimethylammonio)acetate, obtained from Panzeri s.r.l. Their chemical structures are reported in Fig. 9.

### Chemical treatments

For each of the three different fabric softeners, different experimental conditions were tested: concentrations in the range 10–200 mg/L, temperatures between 25 °C and 60 °C and reaction times ranging from 15 to 60 min. The experimental procedure involved the use of 1 gr of fiber, carefully washed and combed after the extraction, which is soaked and stirred in 75 mL of aqueous solution containing the softening agent. Considering the weight of fiber dipped into the treating solution/dispersion, the percentage of softener used, in terms of o.w.f. (on the weight of fiber), goes from 0.15 up to 1.5%. The properties of the treated fibers were compared to those of the raw samples (blank samples), obtained by soaking and stirring 1 gr of fiber in 75 mL of pure water, thus without any softening agent, under the same conditions of the treated samples. The detailed experimental conditions were reported in Table S1. At the end of the treatment the fiber was washed with pure water, wringed out and left to dry naturally at room temperature for 1 week.

### Wettability

A modification of the du Noüy ring method was used to evaluate the water wettability of the different fibers. The zero-contact angle platinum ring, generally used as a probe for measuring the pull forces exerted on it at the liquid/gas interface, has been replaced by probes made by the cellulose fibers. Specifically, the probes were prepared by spinning 10 individual fibers to form yarns of 6 cm length with 2.5 twist/cm. The pull force (mN) was then measured as a function of time, at 26 °C, and the maximum pull force was recorded, after an equilibration time of about 60 s.

Measurements were performed by a TD1 Lauda Tensiometer equipped with a Lauda E100 thermostatic bath with a sensitivity of 0.1 mN/m. All the values measured on the treated fibers were referenced to the average value of the raw fiber, which was then used as the reference probe with zero tension.

Static contact angles to deionized water were measured with a CAM 200 contact angle meter (KSV Instruments LTD, Helsinki,Finland) at room temperature. A drop (5 μL) of deionized water was put onto the sample surface by a micropipette, and measurements were carried out by setting the tangents on both visible edges of the droplet contact angle. Water contact angle (WCA) measurements were performed on fiber disks made by weighing 5 mg of fiber pressed by a KBr pellet. The contact angle was measured immediately after the deposition of the drop of water onto the disk surface. All experiments were carried out at room temperature (22 ± 1 °C) and the contact angle was measured in triplicate and a mean value was obtained for each films^48,49^. The images were processed by ImageJ free software using the contact angle plugin^50^. The program then fits the profile of the drop and calculates the contact angle using the sphere approximation or the ellipse approximation.

### Determination of the linear mass density

This was determined in terms of tex, i.e., grams of fiber per km, by weighing a known length of fiber.

### Powder X-ray diffraction (PXRD) analysis

PXRD patterns were acquired on a Bruker D2-Phaser equipped with a Cu Kα radiation (λ = 1.5418 A) and a Lynxeye detector, at 30 kV and 10 mA, with step size of 0.01° and step time of 2.5 s (acquisition time 10,000 s), over an angular range of 10–50° 2θ. The fibers as they are, were placed on the sample holder and gently pressed with a weight. In order to determine the Crystallinity Index (CI) of the cellulosic samples, the obtained powder patterns, after the subtraction of a blank run including the sample holder, were deconvoluted by applying Voigt fitting procedure^51^ using Origin software. Iterations were repeated until a R2 value of 0.998 was reached. CI was calculated according to the following equation:

2 $$ CI = \frac{{A_{cr} }}{{A_{tot} }} \times 100 $$

where $A_{cr}$ represents the area under the crystalline peaks and $A_{tot}$ is the total area under the PXRD pattern.

### Thermogravimetric analysis (TGA)

TGA was performed on a Perkin Elmer Pyris 6 Thermogravimetric Analyzer. Approximately 2 mg of each sample was placed in an alumina crucible and heated from 30 °C to 800 °C, at a heating rate of 10 °C min^−1^, under a dry nitrogen atmosphere. The DTG curves here reported, which provide the percentage weight loss rate, were obtained from the first derivative of the TG profiles.

### Optical microscopy

Optical microscopy was performed with a Leitz Laborlux 12 POL. The samples were placed between two glass slides and images were recorded at room temperature.

### Scanning electron microscopy (SEM)

A LEO 420 scanning electron microscope (SEM, Zeiss), operating at 15 kV, was used to observe the morphological features of pristine and treated fibers. The fibers were mounted onto aluminium specimen stubs using double-sided adhesive carbon tables and then gold-coated by sputtering (Agar Sputter coater) to avoid electrostatic charge and to improve image resolution. Fiber diameter was measured by the Leo UIF software by selecting three different sample areas of the acquired image, with dimensions (597 × 356) μm^2^ and by measuring the diameter of 20 fibers per each sample area. Statistical analysis was performed by the Origin Pro software (2019). First, the normal distribution of data was tested (Normality test) and then the Analysis of Variance (ANOVA) test was used for mean comparison among the samples using the Tukey Test.

### Three-point bending test

Dynamic mechanical analysis (DMA) measurements were carried out on a Metravib DMA/25 analyzer equipped with a three-point bending geometry. Strain sweep experiments were collected by subjecting the samples to a frequency of amplitude of 1 Hz in the range between 10^−5^ and 3 × 10^−3^ m displacement with a gauge length of 6.5 cm. A periodic sinusoidal displacement was applied to the sample and the resultant force was measured.

The three-point bending test provides values for the modulus of elasticity in bending. It was performed at 25 °C on yarns of 20 tex prepared by spinning 5 individual fibers with 2.5 twist/cm. The experiment records the elastic E’(ω) and viscous E’’(ω) moduli as a function of an increase in the applied deformation (displacement). E′(ω) is the in-phase (or storage) component and E″(ω) is the out-of-phase (or loss) component. E′(ω) is a measure of the reversible elastic energy, while E″(ω) represents the irreversible viscous dissipation of the mechanical energy^52^.

### Plots, statistical analysis and fitting

All plots, the statistical analysis of the raw data and the fitting procedures were performed by Origin, Version *2019b*. OriginLab Corporation, Northampton, MA, USA.

## Supplementary Information

Supplementary Information

## References

[1] Mokhtari F, Salehi M, Zamani F, Hajiani F, Zeighami F, Latifi M (2016). Advances in electrospinning: the production and application of nanofibres and nanofibrous structures. Text. Prog..

[2] Maiti S, Das D, Sen K (2017). Flexible non-metallic electro-conductive textiles. Text. Prog..

[3] Paone E, Beneduci A, Corrente GA, Malara A, Mauriello F (2020). Hydrogenolysis of aromatic ethers under lignin-first conditions. Mol. Cat..

[4] Fasolini A, Cucciniello R, Paone E, Mauriello F, Tabanelli T (2019). A short overview on the hydrogen production via aqueous phase reforming (APR) of cellulose, C6–C5 sugars and polyols. Catalysts.

[5] Xu C, Paone E, Rodríguez-Padrón D, Luque R, Mauriello F (2020). Reductive catalytic routes towards sustainable production of hydrogen, fuels and chemicals from biomass derived polyols. Renew. Sustain. Energy Rev..

[6] Tilman D, Cassman KG, Matson PA, Naylor R, Polasky S (2002). Agricultural sustainability and intensive production practices. Nature.

[7] Reddy N, Yang Y (2005). Properties and potential applications of natural cellulose fibers from Cornhusks. Green. Chem..

[8] Arias F, Beneduci A, Chidichimo F, Furia E, Straface S (2017). Study of the adsorption of mercury (II) on lignocellulosic materials under static and dynamic conditions. Chemosphere.

[9] Tursi A, Beneduci A, Chidichimo F, De Vietro N, Chidichimo G (2018). Remediation of hydrocarbons polluted water by hydrophobic functionalized cellulose. Chemosphere.

[10] Tursi A, Chatzisymeon E, Chidichimo F, Beneduci A, Chidichimo G (2018). Removal of endocrine disrupting chemicals from water: adsorption of bisphenol-A by biobased hydrophobic functionalized cellulose. Int. J. Environ. Res. Public Health.

[11] Tursi A, De Vietro N, Beneduci A, Milella A, Chidichimo F, Fracassi F, Chidichimo G (2019). Low pressure plasma functionalized cellulose fiber for the remediation of petroleum hydrocarbons polluted water. J. Hazard. Mater..

[12] De Vietro N, Tursi A, Beneduci A, Chidichimo F, Milella A, Chatzisymeon E, Fracassi F, Chidichimo G (2019). Photocatalytic inactivation of *Escherichia coli* bacteria in water using low pressure plasma deposited TiO_2_ cellulose fabric. Photochem. Photobiol. Sci..

[13] Angelini LG, Lazzeri A, Levita G, Fontanelli D, Bozzi C (2000). Ramie (*Boehmeria nivea* (L.) Gaud.) and Spanish broom (*Spartium junceum* L.) fibres for composite materials: agronomical aspects, morphology and mechanical properties. Ind. Crops Prod..

[14] Chidichimo G, Aloise A, Beneduci A, De Rango A, Pingitore G, Furgiuele F, Valentino P (2016). Polyurethanes reinforced with spartium junceum fibers. Polym. Compos..

[15] Angelini L. G., Tavarini S. & Foschi L. Spanish broom (*Spartium junceum* L.) as new fiber for biocomposites: the effect of crop age and microbial retting on fiber quality. Conference Papers in Materials Science 2013, 274359. 10.1155/2013/274359

[16] Cerchiara T, Chidichimo G, Gallucci MC, Vuono D (2010). Effects of extraction methods on the morphology and physico-chemical properties of Spanish Broom (*Spartium junceum* L.) fibres. Fibres Text. East. Eur..

[17] Cerchiara T, Chidichimo G, Ragusa M, Belsito E, Liguori A, Arioli A (2010). Characterization and utilization of Spanish Broom (*Spartium junceum* L.) seed oil. Ind. Crop. Prod..

[18] Gabriele B, Cerchiara T, Salerno G, Chidichimo G, Vetere MV, Alampi C, Gallucci MC, Conidi C, Cassano A (2010). A new physical–chemical process for the efficient production of cellulose fibers from Spanish broom (*Spartium junceum* L.). Bioresour. Technol..

[19] Segal L, Nelson ML, Conrad CM (1951). Experiments on the reduction of the crystallinity of cotton cellulose. J. Phys. Chem..

[20] Zhang YHP, Lynd LR (2004). Toward an aggregated understanding of enzymatic hydrolysis of cellulose: noncomplexed cellulase systems. Biotechnol. Bioeng..

[21] Igarashi T, Morita N, Okamoto Y, Nakamura K (2016). Elucidation of softening mechanism in rinse cycle fabric softeners. Part 1: effect of hydrogen bonding. J. Surfact. Deterg..

[22] Igarashi T, Nakamura K, Hoshi M, Hara T, Kojima H, Itou M, Ikeda R, Okamoto Y (2016). Elucidation of softening mechanism in rinse-cycle fabric softeners. Part 2: uneven adsorption-the key phenomenon to the effect of fabric softeners. J. Surfactants Deterg..

[23] French AD (2014). Idealized powder diffraction patterns for cellulose polymorphs. Cellulose.

[24] Kovačević Z, Vukušić SB, Zimniewska M (2012). Comparison of Spanish broom (*Spartium Junceum* L.) and flax (Linumusitatissimum) fibre. Text. Res. J..

[25] Totolin MI, Vasile C, Tibirna CM, Popescu MC (2008). Grafting of Spanish broom (Spartium Junceum) fibers with fatty acids under cold plasma conditions. Cellul. Chem. Technol..

[26] Basheva ES, Danov KD, Radulova GM, Kralchevsky PA, Xu H, Ung YW, Petkov JT (2019). Properties of the micelles of sulfonated methyl esters determined from the stepwise thinning of foam films and by rheological measurements. J. Colloid Interface Sci..

[27] Dhar N, Au D, Berry RC, Tam KC (2012). Interactions of nanocrystalline cellulose with an oppositely charged surfactant in aqueous medium. Colloids Surf. A Physicochem. Eng. Aspects.

[28] Ghoreishi SM, Behpour M, Shabani-Nooshabadi M (2009). Interaction of anionic azo dye and TTAB—cationic surfactant. J. Braz. Chem. Soc..

[29] Yezer BA, Khair AS, Sides PJ, Prieve DC (2016). Determination of charge carrier concentration in doped nonpolar liquids by impedance spectroscopy in the presence of charge adsorption. J. Colloid Interface Sci..

[30] Alila S, Boufi S, Belgacem MN, Beneventi D (2005). Adsorption of a cationic surfactant onto cellulosic fibers I. Surface charge effects. Langmuir.

[31] Brinatti C, Huang J, Berry RM, Tam KC, Loh W (2016). Structural and energetic studies on the interaction of cationic surfactants and cellulose nanocrystals. Langmuir.

[32] Aloulou F, Boufi S, Belgacem N, Gandini A (2004). Adsorption of cationic surfactants and subsequent adsolubilization of organic compounds onto cellulose fibers. Colloids Polym. Sci..

[33] Khan AS, Man Z, Bustam MA, Kait CF, Khan MI, Muhammad N, Nasrullah A, Ullah Z, Ahmad P (2016). Impact of ball-milling pretreatment on pyrolysis behavior and kinetics of crystalline cellulose. Waste Biomass.

[34] Shafizadeh F, Bradbury AGW (1979). Thermal degradation of cellulose in air and nitrogen at low temperatures. J. Appl. Polym. Sci..

[35] Abidi N, Hequet E, Ethridge D (2007). Thermogravimetric analysis of cotton fibers: relationships with maturity and fineness. J. Appl. Polym. Sci..

[36] Price D, Horrocks AR, Akalin M, Faroq AA (1997). Influence of flame retardants on the mechanism of pyrolysis of cotton (cellulose) fabrics in air. J. Anal. Appl. Pyrol..

[37] Hideno A (2016). Comparison of the thermal degradation properties of crystalline and amorphous cellulose, as well as treated lignocellulosic biomass. BioRes.

[38] Brígida AIS, Calado VMA, Gonςalves LRB, Coelho MAZ (2010). Effect of chemical treatments on properties of green coconut fiber. Carbohydr. Polym..

[39] Scarpelli F, Crispini A, Giorno E, Marchetti F, Pettinari R, Di Nicola C, De Santo MP, Fuoco E, Berardi R, Alfano P, Caputo P, Policastro D, Oliviero Rossi C, Aiello I (2020). Preparation and characterization of silver(I) ethylcellulose thin films as potential food packaging materials. ChemPlusChem.

[40] Harkins WD, Jordan HF (1930). A method for the determination of surface and interfacial tension from the maximum pull on a ring. J. Am. Chem. Soc..

[41] Khan, M. I., & Islam, M. R. Enhanced oil recovery (EOR) operations in the petroleum engineering handbook: sustainable operations (2007).

[42] Drelich J., Fang C. & White C. L. Measurement of interfacial tension in fluid-fluid systems. Encyclopedia of Surface and Colloid Science, 3152–3166 (Marcel Dekker, Inc., 2002)

[43] Bao Y, Qian H, Lu Z, Cui S (2015). Revealing the hydrophobicity of natural cellulose by single-molecule experiments. Macromolecules.

[44] Qui Y (2002). Analysis of energy dissipation in twisted fiber bundles under cyclic tensile loading. Textile Res. J..

[45] Jeddy AA, Nosraty H, Taheri Otaghsara MR, Karimi M (2007). A comparative study of the tensile fatigue behavior of cotton-polyester blended yarn by cyclic loading. J. Elastomers Plast..

[46] Olofsson, B., & Gralen, N. Measurement of friction between single fibers; v. frictional properties of viscose rayon stample fibers. *Text. Res. J.* 467–476 (1950).

[47] Obendorf SK, Dixit V, Woo DJ (2009). Microscopy study of distribution of fabrics softener on cotton fabric. J. Surfact. Deterg..

[48] Oliviero Rossi C, Caputo P, Baldino N, Lupi FR, Miriello D, Angelico R (2016). Effects of adhesion promoters on the contact angle of bitumen-aggregate interface. Int. J. Adhes. Adhes..

[49] Oliviero Rossi C, Caputo P, Baldino N, Ildyko Szerb E, Teltayev B (2017). Quantitative evaluation of organosilane-based adhesion promoter effect on bitumen-aggregate bond by contact angle test. Int. J. Adhes. Adhes..

[50] Lamour G, Hamraoui A, Buvailo A, Xing Y, Keuleyan S, Prakash V, Eftekhari-Bafrooei A, Borguet E (2010). Contact angle measurements using a simplified experimental setup. J. Chem. Educ..

[51] Park S, Baker JO, Himmel ME, Parilla PA, Johnson DK (2010). Cellulose crystallinity index: measurement techniques and their impact on interpreting cellulase performance. Biotechnol. Biofuels.

[52] Filippelli L, Gentile L, Oliviero RC, Ranieri GA, Antunes F (2013). Structural change of bitumen in the recycling process by using rheology and NMR. Ind. Eng. Chem. Res..

