# Supplementary material for: Chemical–physical and dynamical–mechanical characterization on Spartium junceum L. cellulosic fiber treated with softener agents: a preliminary investigation
Source: Sci Rep. 2021 Jan 8;11:35. doi: 10.1038/s41598-020-79568-5 (PMC7794538; doi:10.1038/s41598-020-79568-5)
Supplement: Supplementary file 1 — Supplementary Information [file 41598_2020_79568_MOESM1_ESM.docx]

**Supporting Information for**

**Chemical-physical and dynamical-mechanical characterization on *Spartium junceum* L. cellulosic fiber treated with softener agents: a preliminary investigation**

**Giuseppina Anna Corrente, Francesca Scarpelli, Paolino Caputo, Cesare Oliviero Rossi, Alessandra Crispini, Giuseppe Chidichimo, Amerigo Beneduci***

*Department of Chemistry and Chemical Technologies, University of Calabria, Via P. Bucci, Cubo15D, 87036 Arcavacata di Rende (CS), Italy.*

**Table S1** Experimental conditions used for each softener on the cellulosic fiber

|  | Samples | Concentration (mg/L) | Temperature (°C) | Time  (min) |
| --- | --- | --- | --- | --- |
| TTAB | a | 200 | r.t. | 60 |
|  | b | 100 | r.t. | 15 |
|  | c |  |  | 30 |
|  | d |  |  | 60 |
|  | e | 100 | 40 | 15 |
|  | f |  |  | 30 |
|  | g |  |  | 60 |
|  | h | 50 | r.t. | 15 |
|  | i |  |  | 30 |
|  | l |  |  | 60 |
|  | m | 10 | r.t. | 15 |
|  | n |  |  | 30 |
|  | o |  |  | 60 |
| LP | a | 200 | r.t. | 15 |
|  | b |  |  | 30 |
|  | c |  |  | 60 |
|  | d | 200 | 60 | 60 |
|  | e | 100 | r.t. | 60 |
| LS | a | 200 | r.t. | 15 |
|  | b |  |  | 30 |
|  | c |  |  | 60 |
|  | d | 200 | 60 | 60 |
|  | e | 100 | r.t. | 60 |

**Table S2** Thermogravimetric data of raw fiber and TTABm, LPb and LSb samples.

| Sample | Transition | Transition temperature range (°C) | Inflection point (peak max) (°C) | Onset (°C) | Weight loss (%) |
| --- | --- | --- | --- | --- | --- |
| raw fiber | 1 | 30-110 | 60 | \ | \ |
|  | 2 | 256-407 | 365,4 | 340,7 | 53,7 |
|  | 3 | 407-527 | 483,9 | 459,2 | 20,9 |
| TTABm | 1 | 30-110 | 60 | \ | \ |
|  | 2 | 268-409 | 365,3 | 350 | 57,8 |
|  | 3 | 409-531 | 488,1 | 462,7 | 16,2 |
| LPb | 1 | 30-110 | 60 | \ | \ |
|  | 2 | 261-400 | 365,3 | 346,7 | 55,2 |
|  | 3 | 400-523 | 459,6 | 448,2 | 20,6 |
| LSb | 1 | 30-110 | 60 | \ | \ |
|  | 2 | 264-398 | 363 | 345,2 | 54,4 |
|  | 3 | 398-525 | 472,8 | 459,6 | 18,8 |
